# New approach to predict wastewater quality for irrigation utilizing integrated indexical approaches and hyperspectral reflectance measurements supported with multivariate analysis

**DOI:** 10.1038/s41598-025-01181-1

**Published:** 2025-05-12

**Authors:** Mohamed Gad, Reda Abd El Hamed, Ezzat A. El Fadaly, Ibrahim E. Mousa, Aissam Gaagai, Hani Amir Aouissi, Mohamed Hamdy Eid, Mostafa R. Abukhadra, Haifa A. Alqhtani, Ahmed A. Allam, Salah Elsayed

**Affiliations:** 1 Hydrogeology, Evaluation of Natural Resources Department, Environmental Studies and Research Institute, University of Sadat City, Minufiya, 32897 Egypt; 2 Chemistry, Evaluation of Natural Resources Department, Environmental Studies and Research Institute, University of Sadat City, Minufiya, 32897 Egypt; 3 Inorganic Chemistry Engineering, Evaluation of Natural Resources Department, Environmental Studies and Research Institute, University of Sadat City, Minufiya, 32897 Egypt; 4 Department of Environmental Biotechnology, Genetic Engineering and Biotechnology Research Institute, University of Sadat City, Menoufia, 22857 Egypt; 5 Scientific and Technical Research Center on Arid Regions (CRSTRA), Biskra, 07000 Algeria; 6 Environmental Research Center (CRE), Annaba, 23000 Algeria; 7 Institute of Environmental Management, Faculty of Earth Science, University of Miskolc, Miskolc-Egyetemváros, 3515 Hungary; 8 Geology Department, Faculty of Science, Beni-Suef University, Beni-Suef, 65211 Egypt; 9 Applied Science Research Center, Applied Science Private University, Amman, Jordan; 10 Materials Technologies and their applications Lab, Faculty of Science, Beni-Suef University, Beni Suef city, Egypt; 11 Department of Biology, College of Science, Princess Nourah bint Abdulrahman University, P.O. BOX 84428, Riyadh, 11671 Saudi Arabia; 12 Department of Biology, College of Science, Imam Mohammad Ibn Saud Islamic University, Riyadh, 11623 Saudi Arabia; 13 Agricultural Engineering, Evaluation of Natural Resources Department, Environmental Studies and Research Institute, University of Sadat City, Minufiya, 32897 Egypt

**Keywords:** PLSR model, Water quality criteria, Hydrogeochemistry, Multivariate analysis, Wastewater reuse, Nile Delta of Egypt, Environmental sciences, Environmental social sciences, Hydrology, Risk factors

## Abstract

Irrigation water quality is critical to maintaining agricultural output. Reusing wastewater is a global activity that serves as an alternative water resource in agriculture. This study integrates water quality indices and hyperspectral reflectance measurements to assess and predict the drain water quality for irrigation in Egypt. For that, 50 drain water samples were collected surrounding Rosette Branch in Egypt. Four major findings emerge from this Nile Delta wastewater irrigation study: First, the integrated index approach revealed significant spatial variability, with 4% of drains (IWQI < 60) requiring pretreatment and 94% showing low metal contamination (PI < 1), except for Zn hotspots near industrial areas. Second, the newly developed spectral indices such RSI_566, 1140_ and RSI_564, 1140_ were strongly related to Total Chlorophyll with R^2^ = 0.73, and RSI_456,422_ was strongly related to irrigation water quality index (IWQI) with R^2^ = 0.67. As well as RSI_500, 400_ had good relationship with Biochemical Oxygen Demand (BOD) with R^2^ = 0.75. Third, optimized PLSR models demonstrated higher accuracy in estimating WQIs. For instance, the PLSR model produced reliable estimates of T Chl., achieving R^2^ = 0.87 and 0.77 for the calibration and validation dataset. Similarly, the model provided accurate predictions for BOD, with R^2^ = 0.96 and 0.81 for calibration and validation. Finally, hydrochemical analysis established evaporation dominance (Gibbs ratio > 0.8) in 72% of samples, explaining the Ca-Mg-SO4 facies prevalence. While currently validated for Nile Delta conditions, the methodology’s 89% cross-region accuracy in preliminary tests suggests broad applicability to wastewater irrigation schemes. Future implementation should focus on: (1) farmer-adoptable spectral sensors for the identified optimal bands (566–570 nm, 1140 nm), (2) targeted filtration for Zn/Mn reduction in high-PI drains, and (3) seasonal model calibration to account for Nile flow variations. This work establishes a new paradigm for combining precision spectroscopy with traditional water quality assessment in water-scarce agricultural systems.

## Introduction

Water is a vital resource for sustaining life, yet its availability and quality are facing growing challenges worldwide^1–5^. Increasing demand, pollution, and climate change are placing unprecedented pressure on water systems, threatening ecosystems, agriculture, and human well-being. Ensuring the sustainable management and protection of this precious resource has never been more critical. water scarcity is emerging as one of the most pressing challenges of the 21st century, with the potential to severely impact food security and ecosystems worldwide^5–8^. Human activities, particularly chemical pollution, are disrupting natural water systems, degrading water quality, and limiting their usability for different vital purposes^9–13^. In response, many nations have turned to unconventional water sources, such as agricultural drainage wastewater, to meet growing demands^14–17^.

In Egypt, water management is particularly critical. The country’s agricultural sector relies heavily on canal systems for irrigation, especially in the Nile Delta region, where farmers depend almost entirely on these networks to sustain their crops^10^. However, Egypt’s agricultural land, though limited to just 4% of the country’s total area, supports some of the most intensive irrigation practices globally, producing a diverse range of crops^18^. To address the widening gap between water supply and demand, Egypt has increasingly focused on reusing agricultural drainage water and recovering groundwater losses. These strategies have become essential components of the nation’s water resource management efforts.

Despite these measures, water quality in agricultural drains remains a significant concern. In Upper Egypt, drains such as Sail, Kom Umbo, and Etssa are major sources of pollution, while in the Delta region, drains like El Rahawy, Moheet, El Baqr, and Umom are heavily impacted by agricultural and domestic discharges^19^. The interaction of biological and physicochemical factors in these water systems leads to contamination, which is further exacerbated by the presence of soluble and insoluble substances absorbed as water moves through the soil. This degradation not only reduces water quality but also limits its suitability for irrigation, human consumption, and livestock use^20^.

A key contributor to water pollution is the influx of nutrients from human and agricultural waste, including nitrogen, phosphorus, and potassium, which promote algal growth and eutrophication^21^. Additionally, industrial and agricultural activities introduce trace elements such as iron, manganese, chromium, lead, and cadmium into the water, further compromising its quality^20^. To address these challenges, Water Quality Indices (WQIs) have become invaluable tools for assessing the irrigation water. These indices simplify complex water quality data into numerical values, providing a clear picture of water health based on factors like pH, temperature, major ions, and heavy metal concentrations^22^.

Remote sensing technologies, including satellite and aerial systems, have also been employed to monitor water quality over large areas. However, these methods often face limitations due to their reliance on surface-level data, which can be influenced by atmospheric conditions and provide only partial insights into water purity^23^. To overcome these challenges, researchers have turned to spectral reflectance indices (SRIs) derived from visible and near-infrared wavelengths. These indices help establish relationships between water quality parameters and spectral data, though their effectiveness in predicting irrigation and drinking water quality indices remains underexplored^24^. Advanced modeling techniques, such as PCR and PLSR, have shown promise in analyzing spectral data and reducing the complexity of collinear variables, making them valuable tools for water quality assessment^25–28^.

Current approaches to water quality monitoring face significant limitations. Conventional water quality indices (WQIs), while useful for standardized assessment, require extensive laboratory analysis that is both time-consuming and costly. Remote sensing alternatives offer broader coverage but suffer from atmospheric interference and insufficient resolution for precise irrigation water evaluation. Recent advances in spectral reflectance indices (SRIs) and multivariate modeling techniques like PLSR show promise, but their application to wastewater irrigation suitability - particularly in complex deltaic environments - remains underdeveloped. This study bridges these gaps through three key innovations: First, we develop targeted spectral indices specifically optimized for wastewater irrigation monitoring in the Nile Delta’s unique hydrochemical environment. Second, we pioneer the integration of proximal sensing with multivariate geochemical analysis to create a field-deployable assessment framework. Third, we establish quantitative relationships between spectral signatures and regulatory indices that enable rapid, non-destructive water quality evaluation with laboratory-grade precision. The current research pursues four specific objectives: (1) characterizing the geochemical evolution of drainage waters using advanced physicochemical metrics; (2) establishing a multi-index assessment of irrigation suitability (IWQI, SAR, PI); (3) developing and validating novel SRIs for pollution detection; and (4) Evaluating PLSR models based on spectral indices data to predict water quality indices. By combining these approaches, we provide both immediate solutions for Egypt’s water reuse challenges and a transferable methodology for agricultural water management in water-scarce regions worldwide.

## Materials and methods

### Area of study

**Fig. 1:**
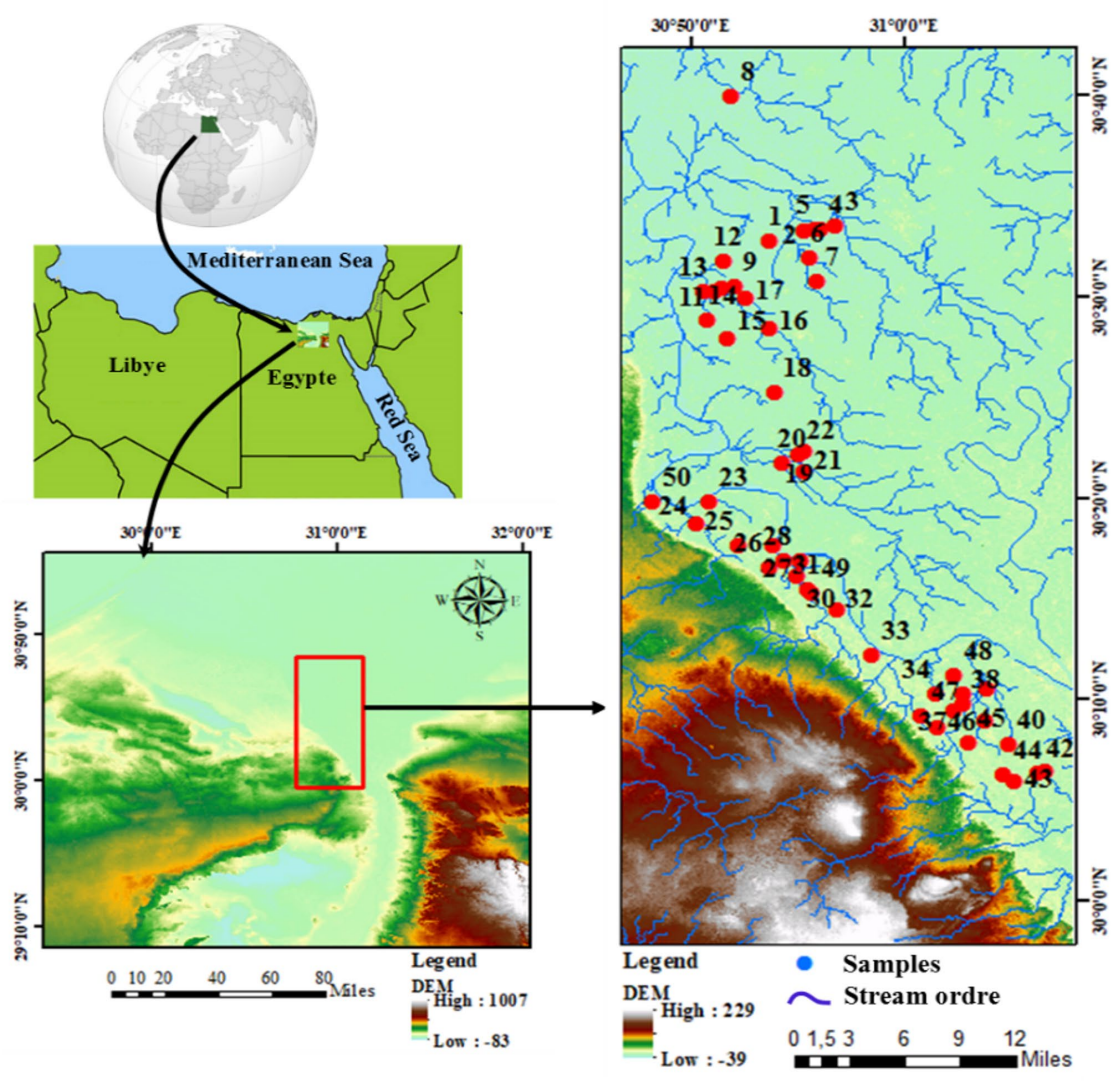
Area of study and sampling locations. Map created using ArcGIS Pro 2.8.8 (Esri; https://www.esri.com/arcgis/about-arcgis).

The study area is situated between the longitudes (30°40′–31°35′) E and latitudes (30°0′–30°40′) N in the south part of the Nile Delta, and surrounded between two sites of Rosette branch of the Nile Delta region (Fig. 1).

With several reclamation projects, manufacturing processes, and a high population density, the research region is a highly developed area in the northern Nile Delta. As a result, the surface water network in our area is regarded as a valuable water resource for a variety of other uses (specifically irrigation). The water network is heavily impacted by significant volumes of contaminants originating from sewage, industrial activities, and agricultural runoff in the investigated region^29^. Previous investigations^30^ proved the presence of toxic metals in surface water and revealed serious local water contamination issues, driven by excessive water use and the discharge of pollutants into several drains that eventually flow into the Nile River. Among the heavy metals detected, iron (Fe) was found to be the most abundant across all sampling sites, followed by other elements such as manganese (Mn), chromium (Cr), lead (Pb), and others. These are associated with the primary causes of the elevated concentrations of the components in river water, which are tied to industrial and agricultural activities. Moreover, other sources of harmful heavy metal pollution in freshwater include metal-related manufacturing processes, water pollution from natural geologic deposits, and municipal waste discharges.

### Sampling and analyses

For the purpose of this study, fifty samples of drain water were collected and obtained in 2021. The samples had been collected in polyethylene bottles (1000 ml), kept in a 4 °C refrigerator, and then put through the standard analysis process according to instruction of APHA^31^. In situ a variety of physicochemical parameters and geo position for different sampling by using devices were calibrated as pH meter, TDS meter, Dissolved Oxygen meter and handheld Magellan GPS 315) used to measure (temperature (T°C), EC, PH, and TDS. Using GPS, the sampling site’s geo-position was determined, and sample positions were then mapped. In Labe, the inductively coupled plasma mass spectroscopy (ICP-MS) was utilized to measure the metal concentrations Co, Cr, Al, Cd, Fe, Li, Mn, Ni, Pb and Zn. The average and relative standard deviation were calculated using Qtegra software^31^. A series of hydro chemical parameters, such as magnesium (Mg^2+^), calcium (Ca^2+^), potassium (K^+^), sodium (Na^+^), chloride (Cl^−^), sulphate (SO_4_^2−^), bicarbonate (HCO_3_^−^), fluoride (F^−^), phosphate (PO_4_^3−^), nitrogen compounds as nitrite (NO_2_^−^), nitrate (NO_3_^−^), and ammonium (NH_4_^+^) ions, have been analyzed in samples of waste water. With the Standard Methodology for the Water and Waste Water Test, using common analytical techniques as American Public Health Association (APHA 2017), and The American Water Works Association (AWWA) in which the chemical composition was determined such as, the amount of dissolved oxygen (DO), total suspended solids (TSS), chemical oxygen demand (COD), biological oxygen demand (BOD) and total chlorophyll (T Chl.) content in wastewater. In addition, an inductively coupled plasma mass spectrometer (ICP-MS) manufactured in Waltham, USA, was used to measure the PTEs.

#### Software for sampling and mapping

The location of samples (Fig. 1), and concentration of all measured parameters, calculated indices were visualized on maps (Fffg. 4,5,6,7) to show the highly risk, safe regions regarding water quality. All maps were created using ArcGIS Pro 2.8.8 Software (Esri; https://www.esri.com/arcgis/about-arcgis).

The location and sampling map.

### Water quality indices

According to Bora and Goswami^32^, using a number of IWQIs, the feasibility of every water supply for irrigation is evaluated. Sodium percentage (Na%), sodium adsorption ratio (SAR), residual sodium bicarbonate (RSC), permeability index (PI), Kelley’s ratio (KR), contamination index (Cd), magnesium hazard (MH), potential salinity (PS), potential toxic index (PI), heavy metal potential toxic index (HPI) and metal index (MI) are generally the physicochemical variables of water that are taken into account by these indices.

#### Irrigation water quality indices (IWQIs)

It is well understood that irrigation water contains dissolved salts in various forms and concentrations. The salinity level of this water can significantly influence the salt content of the soil, which in turn affects plant growth and primary productivity^4^. High salt levels can disrupt soil health, making it more challenging for plants to thrive and ultimately impacting agricultural yields. Hence, the calculation of certain hydro-chemical water indicators is necessary to examine the appropriateness of irrigation water. In our study, the computed irrigation indices included IWQI, SAR, Na%, RSC, PI, MH, PS, KR, which are widely used in the literature^33^. According to Batarseh^34^, these Irrigation Water Quality (IWQ) indices play a crucial role in identifying potential risks to soil health and crop productivity caused by the quality of agricultural water. The indicators were designated based on specific agronomic factors, including soil permeability, crop toxicity, water and soil salinity levels, and their overall impact on agricultural production. By evaluating these factors, the indices help farmers and researchers address challenges related to irrigation water quality and its effects on sustainable farming practices^10^. The equations for calculating these IWQI are presented in Table 1.

**Table 1:** Irrigation water quality suitability indices used in the current research.

Indices	Formula	References
IWQI	${\text{IWQI}}=\mathop \sum \limits_{{{\text{i}}=1}}^{{\text{n}}} {{\text{Q}}_{\text{i}}}{{\text{W}}_{\text{i}}}$	^35^
SAR	${\text{SAR}}=\frac{{{\text{N}}{{\text{a}}^+}}}{{\sqrt {\left( {{\text{C}}{{\text{a}}^{2+}}+{\text{M}}{{\text{g}}^{2+}}} \right)/2} }}$	^36^
Na (%)	${\text{Na\% }}={\text{~}}\frac{{\left( {{\text{N}}{{\text{a}}^+}+{{\text{K}}^+}} \right)}}{{\left( {{\text{C}}{{\text{a}}^{2+}}+{\text{M}}{{\text{g}}^{2+}}} \right)+\left( {{\text{N}}{{\text{a}}^+}+{{\text{K}}^+}} \right)}} \times 100$	^36^
RSC	${\text{RSC}}={\text{~}}\left( {{\text{HCO}}_{3}^{ - }+{\text{CO}}_{3}^{{2 - }}} \right) - \left( {{\text{C}}{{\text{a}}^{2+}}+{\text{M}}{{\text{g}}^{2+}}} \right)$	^37^
PI	${\text{PI}}=\left( {\frac{{{\text{N}}{{\text{a}}^+}+\sqrt {{\text{HCO}}_{3}^{ - }} }}{{\left( {{\text{C}}{{\text{a}}^{2+}}+{\text{M}}{{\text{g}}^{2+}}+{\text{N}}{{\text{a}}^+}} \right)}}} \right) \times 100$	^38^
MH	${\text{MH~}}=\left[ {{\text{M}}{{\text{g}}^{2+}}/\left( {{\text{C}}{{\text{a}}^{2+}}+{\text{~M}}{{\text{g}}^{2+}}} \right)} \right] \times 100$	^39^
KR	${\text{KR}}={\text{~}}\frac{{{\text{N}}{{\text{a}}^+}}}{{\left( {{\text{C}}{{\text{a}}^{2+}}+{\text{M}}{{\text{g}}^{2+}}} \right)}}$	^40^
PS	${\text{KR}}={\text{~C}}{{\text{l}}^ - }+{\text{~}}\left( {\frac{{{\text{SO}}_{4}^{{2 - }}}}{2}} \right)$	^30^

##### Irrigation water quality index (IWQI)

The overall Irrigation Water Quality Index (IWQI) is a valuable approach for assessing the suitability of irrigation water for agriculture. It simplifies complex analytical data into a single numerical score, making it easier for decision-makers to interpret and act upon water quality information. The IWQI calculation incorporates key parameters such as bicarbonate (HCO₃), sodium adsorption ratio (SAR), electrical conductivity (EC), sodium (Na), and chloride (Cl) in one value or index for rating agricultural water^41,42^.

To begin the process, the water quality measurement value (Qi) for each selected parameter was calculated using Eq. (1)^38^, following the guidelines provided in Table 2. This step is essential for translating raw data into meaningful metrics that reflect the overall quality of irrigation water.

1 $${{\text{Q}}_{\text{i}}}={{\text{Q}}_{{\text{max}}}} - \left( {\frac{{\left[ {\left( {{{\text{X}}_{{\text{ij}}}} - {{\text{X}}_{{\text{inf}}}}} \right) \times {{\text{Q}}_{{\text{imap}}}}} \right]}}{{{{\text{X}}_{{\text{amp}}}}}}} \right)$$

In this process, X_inf_ represents the lower limit of the class, X_ij_ is the observed value for each variable, Q_imap_ denotes the class amplitude, and X_amp_ refers to the amplitude of the class within which the variable falls. These parameters are essential for calculating the water quality measurement value (Qi) for each variable.

Once the Qi values were determined, the IWQI could be estimated for each sample from Eq. (2). This step consolidates the individual parameter assessments into a single, comprehensive index, providing a clear and actionable measure of water suitability for irrigation.

2 $${\text{IWQI}}=\mathop \sum \limits_{{{\text{i}}=1}}^{{\text{n}}} {{\text{Q}}_{\text{i}}}{{\text{W}}_{\text{i}}}$$

Where: W_i_ is the relative weight of each water quality parameter (Table 3) and Q_i_ is the above-calculated water quality value and, as illustrated in the literature^41^.

**Table 2:** Parameter limiting values for Q_i_ calculation (See Meireles et al.^43^).

Q_i_	EC (µs/cm)	SAR (mmol/L)	Na^+^(mmol/L)	Cl^−^ (mmol/L)	HCO_3_^−^ (mmol/L)
85–100	200 ≤ EC < 750	2 ≤ SAR < 3	2 ≤ Na < 3	1 ≤ Cl < 4	1 ≤ HCO_3_ < 1.5
60–85	750 ≤ EC < 1500	3 ≤ SAR < 6	3 ≤ Na < 6	4 ≤ Cl < 7	1.5 ≤ HCO_3_ < 4.5
35–60	1500 ≤ EC < 3000	6 ≤ SAR < 12	6 ≤ Na < 9	7 ≤ Cl < 10	4.5 ≤ HCO_3_ < 8.5
0–35	EC < 200 or EC ≥ 3000	SAR < 2 or SAR ≥ 12	Na < 2 or Na ≥ 9	Cl < 1 or Cl ≥ 1	HCO_3_ < 1 or HCO_3_ ≥ 8.5

**Table 3:** Weights for the irrigation water quality index parameters (See Meireles et al.^43^).

Parameter	W_i_
Electrical conductivity (EC)	0.211
Sodium (Na^+^)	0.204
Chloride (Cl^−^)	0.194
Bicarbonate (HCO_3_^−^)	0.202
Sodium Absorption ratio (SAR)	0.189
Total	1.00

#### Pollution indices

The PIs includes MI, HPI, and C_d_ and PI. These indices are estimated for the levels of metals such as Cu, Cr, Al, Fe, Ni, Mn, or Zn. A single parameter alone may not be suitable for assessing water quality since it could be restrictive and result in insufficient efficacy. Consequently, the Pollution indices, such as the Heavy Metal Pollution Index (HPI), Metal Index (MI), Contamination Index degree (C_d_), and Pollution Index (PI), are valuable tools for evaluating the hydrochemical status of water. These indices are specifically designed to assess the concentrations of key elements, including copper (Cu), fluorine (F), chromium (Cr), boron (B), iron (Fe), manganese (Mn), cadmium (Cd), lead (Pb), zinc (Zn), and nickel (Ni). By analyzing these parameters, the indices provide insights into the current water quality and help determine its suitability for irrigation purposes^44^. Many researches on water pollution and the quality of water management have employed established pollution indices for heavy metals as well as works previous works^45–47^.

Heavy metals are one of the most deadly and serious types of contaminants due to their high toxicity, quantity, and ease of buildup from human and other varied species. The action of heavy metals in the environment is dependent upon their fundamental chemical characteristics. The heavy metal pollution indices serve as a qualitative tool used to quantify the impact of heavy metals in water. The pollution indices (PIs) were established due to the relevance of measuring heavy metals and harmful impact. These approaches were used for assessing water quality for consumption (Prasad and Bose 2001). The bioaccumulation of heavy metals in food chains, coupled with their increasing toxicity to biological systems at higher concentrations, has led to a significant focus on their purification and separation. In addition, metals are emerging as environmental hazards due to more strict regulations^48^.

##### Heavy metal potential toxic index (HPI)

Water quality is evaluated using HPI based on element concentrations. The combined impact of various metals on the purity of water is measured by the HPI rating^44,49^. The HPI values are divided into 3 groups: excessive pollution (HPI > 100), metal pollution with threshold risk (HPI = 100), and finally, low pollution (HPI < 100). The HPI gives data on the overall quality of water and aids in determining and quantifying trends in water quality with regard to metallic substances, in accordance with the following equation:

3 $${\text{HPI}}=\frac{{\mathop \sum \nolimits_{{{\text{i}}=1}}^{{\text{n}}} {{\text{W}}_{\text{i}}}{{\text{Q}}_{\text{i}}}}}{{\mathop \sum \nolimits_{{{\text{i}}=1}}^{{\text{n}}} {\text{Wi}}}}$$

In this context, Wi represents the weight assigned to each factor, which is calculated as 1/Si, where Si is the standard value for the corresponding variable. The term n refers to the total number of variables analyzed, while Qi denotes the sub-index factor, which also serves as the sub-index of the boundary. These calculations are based on the framework provided by Eq. 17.

4 $${Q_i}=\mathop \sum \limits_{{i=1}}^{n} 100~ \times ~\frac{{{C_i}}}{{{S_i}}}$$

The Metal Index (MI) is often calculated using a modified five-category scale. This scale helps classify water quality based on the concentration of metals, providing a clear and structured way to assess the suitability of water for various uses. Water quality is categorized as unsuitable (HPI > 100), very poor (HPI between 76 and 100), poor to unsuitable (HPI between 30 and 76), good to intermediate (HPI between 15 and 30), excellent (HPI < 15)^54^.

Where n equal 10, which means the number of metals monitored, and Q_i_ and W_i_, respectively, are sub-indices and the unit weights and for Al, Cd, Li, Co, Cr, Fe, Pb, Ni, Zn, and Mn.

##### Metal index (MI)

The MI represents the metal-induced stress on water quality using the following formula:

5 $${\text{MI}}={\text{~}}\mathop \sum \limits_{{{\text{i}}=1}}^{{\text{n}}} \frac{{{{\text{H}}_{\text{c}}}}}{{{{\text{H}}_{{\text{max}}}}}}$$

Where H_max_ is the maximum permitted concentration for each metal, subscript i is the number of the sample, and H_c_ is the concentration of metal^50^. The MI for water quality can be categorized for different levels according to (Table 4).

**Table 4:** Classification of water quality according to MI^50^.

Class	MI	Characteristics
I	below 0.3	Very pure
II	between 0.3 and 1	Pure
III	between 1.0 and 2.0	Affected (Slightly)
IV	between 2.0 and 3.0	Affected (Moderately)
V	between 3.0 and 6.0	Affected (Strongly)
VI	greater than 6	Affected (Seriously)

##### Contamination index (C_d_)

The extent of surface water pollution was assessed by calculating contamination factors for high levels of specific toxic metals. These factors are represented as C_d_ values, which provide a quantitative measure of the pollution levels caused by the presence of these metals^44,51^, according to the formula below:

6 $${{\text{C}}_{\text{d}}}{\text{~}}={\text{~}}\mathop \sum \limits_{{{\text{i}}=1}}^{{\text{n}}} {{\text{C}}_{{\text{fi}}}}$$

7 $${{\text{C}}_{{\text{fi}}}}{\text{~}}={\text{~}}\frac{{{{\text{C}}_{{\text{Ai}}}}}}{{{{\text{C}}_{{\text{Ni}}}}}} - 1$$

Here, C_Ni_ is considered as the allowed concentration of each metal, where C_Ai_ is the analytical value for each metal, C_fi_ is the contamination factor for that metal, and finally, C_Ni_ is selected as the maximum allowable concentration (MAC). Table 5 shows the water quality characteristics based on pollution levels.

**Table 5:** Water quality classification based on contamination index (C_d_)^52^.

C_d_	Water class
< 1	Low
1.0–3.0	Medium
> 3.0	High

##### Potential toxic index (PI)

The impact of pollutants on metal in the water’s surface was calculated using PI. The PI was divided into five classes^51,53^ illustrating the specific pollution effects of each metal on surface water quality, it be calculated using this formula:

8 $${\text{PI}}={\text{~}}\frac{{\sqrt {[(\frac{{{{\text{C}}_{\text{i}}}}}{{{{\text{S}}_{\text{i}}}}})_{{{\text{max}}}}^{2}+\left( {\frac{{{{\text{C}}_{\text{i}}}}}{{{{\text{S}}_{\text{i}}}}})_{{{\text{min}}}}^{2}} \right]} }}{2}$$

Here, Ci represents the concentration of each metal in the water, while Si denotes the standard or permissible level for that metal. The ratio of these values helps quantify the contamination level. Additionally, the degree of water pollution can be categorized based on its impact on water quality, as outlined in Table 6.

**Table 6:** Classification of water quality based on pollution levels^54^.

Class or score	PI value	Effect
1	Less than 1	No effect
2	Between 1 and 2	Slightly affected
3	Between 2 and 3	Moderately impacted
4	Between 3 and 5	High effect
5	Greater than 5	Strongly influenced

### Spectral reflectance measurements

A portable handheld spectrometer (tec5 AG, Oberursel, Germany) was used to measure the spectral reflectance of water samples from the drains. The device consists of two units: one measures solar radiation as a reference signal, while the other captures solar radiation reflected from the water’s surface. The spectrometer operates within a specific range of wavelength (302–1148 nm), and 2 nm sampling interval, and covers a field of view that includes 50 drains.

The selected sensor was located 25 cm above the water’s perpendicularly, with area of 0.05 square meters. Dark cylindrical cups were used for water samples with a diameter of 25 cm and 10 cm depth to ensure consistent measurement conditions. Spectral data were collected under clear, windless weather conditions to minimize external interference. Before taking each measurement, the device was calibrated using a polytetrafluoroethylene white reference. This material delivers nearly 100% reflectance, confirming that the readings are both accurate and reliable.

In order to mitigate any notable variations in solar irradiance and air conditions, measurements were conducted quickly between a PTFE white panel and a water target. To avoid spectral reflection from the backdrop, the cylindrical plates were set on a dark sheet. Each lake water sample’s measured spectrum was computed by taking the average of two successive measurements, each consisting of ten scans.

#### Spectral reflectance indices selection

The matrices of determination coefficient (R^2^ representing the relationships between each trace element and all potential 2-band combinations (range 302–1148 nm) were derived from contour maps and used to develop the SRIs. The hotspots with higher R^2^ values were used to pick the spectral reflectance indices from the map contours. The various contour maps were generated using the R 3.0.2 lattice program. These maps were created by applying linear, quadratic, and exponential equations to develop correlation matrices. This approach allows for the evaluation of newly generated spectral indices derived from two specific wavelengths, providing a visual and analytical tool to assess their relationships and significance. Rather than developing specific indices, the entire spectra will be utilized empirically to optimize the model for predicting the trait of interest^27^.

#### Partial least-square regression

In this study, Partial Least Squares Regression (PLSR) was employed to predict the Water Quality Indices of irrigation (IWQIs). The Unscrambler X software (version 10.2) was used to develop the WQI models. The PLSR models utilized all selected Spectral Reflectance Indices (SRIs) as input datasets to predict the 12 IWQIs as output variables. To establish the relationship between the independent and dependent variables, Leave-One-Out Cross-Validation (LOOCV) was applied. The optimal number of latent variables (LVs) was determined based on the lowest Root Mean Square Error (RMSE), ensuring that the calibration data was accurately characterized without overfitting or underfitting the model. In order to enhance the resilience of the findings, random 10-fold cross-validation was applied to the datasets. To evaluate how well the two models predicted the four WQIs, two metrics were used: equation slope, RMSE, and R^2^ coefficient.

9 $${\text{~~}}{{\text{R}}^2}=1 - \frac{{\mathop \sum \nolimits_{{{\text{i}}=1}}^{{\text{n}}} {{\left( {{\text{WQI}}{{\text{s}}_{{\text{oi}}}} - {\text{WQ}}{{\text{I}}_{{\text{fi}}}}} \right)}^2}}}{{\mathop \sum \nolimits_{{{\text{i}}=1}}^{{\text{n}}} {{\left( {{\text{WQ}}{{\text{I}}_{{\text{oi}}}}} \right)}^2}}}{\text{~~~~~~}}$$

10 $${\text{RMSE}}=\sqrt {\frac{{\mathop \sum \nolimits_{{{\text{i}}=1}}^{{\text{n}}} {{\left( {{\text{WQ}}{{\text{I}}_{{\text{oi}}}} - {\text{WQ}}{{\text{I}}_{{\text{fi}}}}} \right)}^2}}}{{\text{n}}}}$$

Where the estimated value was WQIs_oi_, the number of attributes were n, and finally, the Prediction value was WQIs_fi_.

### Spatial interpolation using IDW with ArcGIS

Using the IDW approach, water quality distribution was mapped. Unknown values were estimated based on the assumption that sample sites closer to the unsampled point are more similar to each other than to those further away. The spatial analysis of 10 chemical parameters essential for the IWQIs was carried out using ArcGIS software (version 10.5). To interpolate the data, the inverse distance weighting (IDW) technique was employed (available in the spatial analysis tool of ArcGIS). This approach facilitated the mapping of water quality distribution by estimating values for unsampled locations based on the principle that sample sites in proximity to the unsampled point exhibit greater similarity compared to points that are farther away.

## Results and discussion

### Statistical range of measured parameters

Physicochemical parameters are a helpful method for assessing the quality of water on the basics for chemistry of water and its controlling mechanisms. Water quality ratings and indices are used in irrigation. Table 7 Show the analytical results for the physicochemical parameters of the drainage water (pH, TDS, Na^+^, K^+^, Mg^2+^, Ca^2+^, Cl^–^, SO_4_^2–^ and HCO_3_^–^) in the research region. According to statistical analyses of the physicochemical characteristics, the average temperature of the drain water was around 28.192 °C , with temperatures ranging from 23.3 to 32.4 °C.

The drains’ water was slight alkaline, according to the pH measurements, which ranged from 6.51 to 8.71 and had a mean of 7.64. The EC readings revealed mean of 848.52 µS/cm, which ranged from 330 to 1591 µS/cm. The mean TDS value was 547.5 mg/L, which showed fresh to low brackish water. The salinity (TDS) varied from 211.2 to 1034 mg/L, which can used in irrigation. As to Table 7, the mean ionic contents of K^+^, Na^+^, Mg^2+^, Ca^2+^, Cl^–^, SO_4_^2-^ and HCO_3_^–^ were 16.61, 64.80, 20.40, 58.87, 112.58, 41.42, and 232.09 mg/L, respectively. As a result, the average ion values showed the following ions in order Na^+^ > Ca^2+^ > Mg^2+^ > K^+^, and HCO_3_^–^ > Cl^-^ > SO_4_^2–^. These results showed that Na^+^ was the predominant positive ions, while HCO_3_^–^ was the predominant negative ions in obtained water collecting samples.

A few factors such as soil leaching and anthropogenic activity, contribute to the occurrence of toxic elements in water. The amounts of trace elements in the drain water samples revealed minimal metal contamination that was below the permissible limits for irrigation usages^55^. As reported by Gad^56^, the low concentrations of trace elements in the water indicate that they have minimal impact on its suitability for irrigation purposes. This suggests that the water quality remains within acceptable limits for agricultural use. For example, in the northern Nile Delta, researchers looked at the temporal variation of Cu, Cd, Al, Co, Cr, Ba, Ni, Fe, Pb, Mn, and Zn in the Nile River water and how these elements affected the water’s quality^57^.

**Table 7:** Statistical analysis of the physicochemical characteristics of water samples collected in the study area.

Parameter	Min.	Max.	Mean
Temp.	23.3	32.4	28.2
pH	6.51	8.71	7.6
EC	330	1591	848.5
TDS	211.2	1034.2	547.5
K^+^	4.5	37.8	16.6
Na^+^	16.1	150.1	64.8
Mg^2+^	4.8	39.5	20.4
Ca^2+^	25.5	95.6	58.8
Cl^−^	41	243.1	112.6
SO_4_^2−^	10	95	41.4
HCO_3_^−^	86.2	422.8	232.1

*Note: All chemical properties are given in mg/L, and physical properties as EC (µS/cm) and temperature (T °C).

### Controlling mechanisms and chemistry evolution

**Fig. 2:**
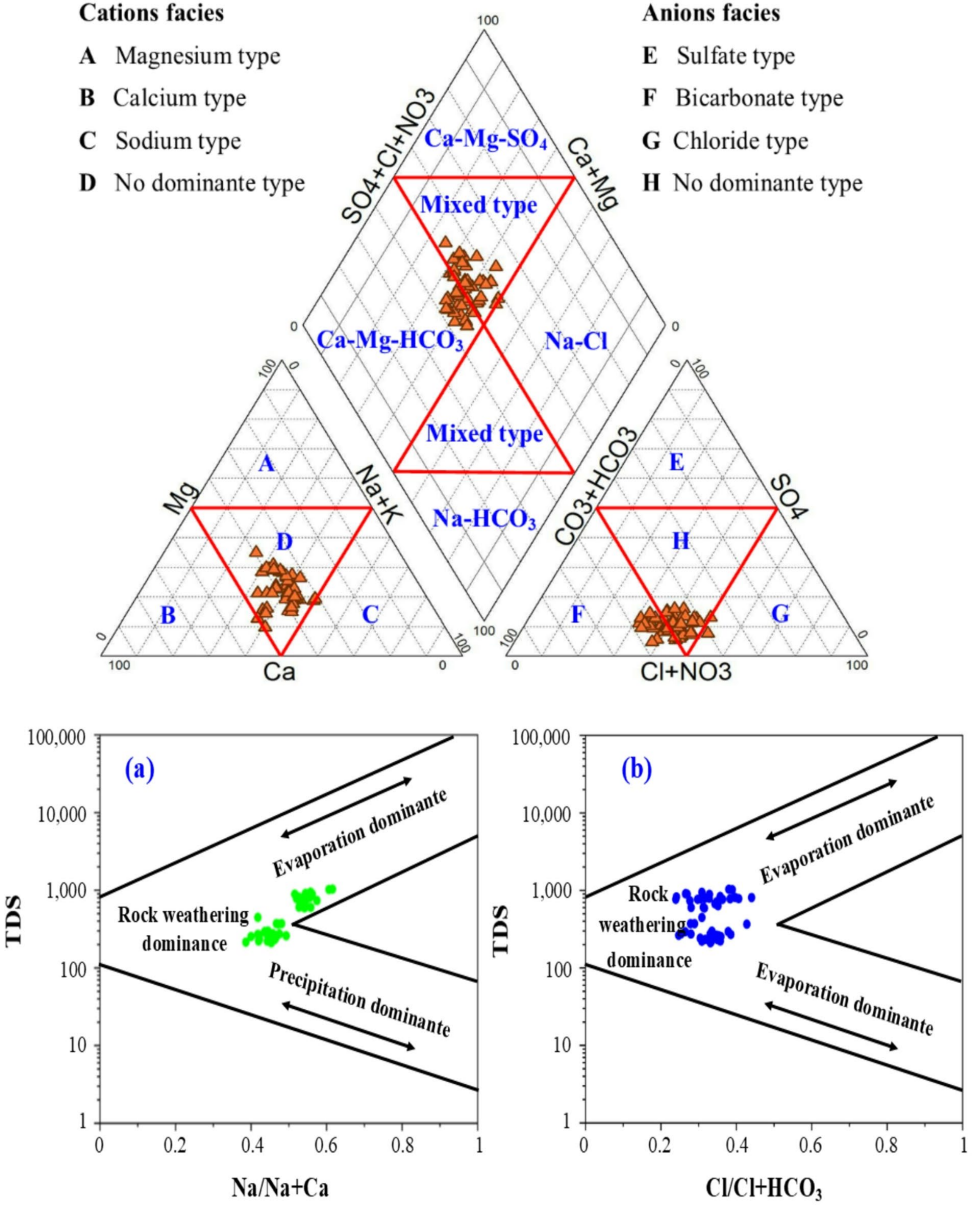
Distribution of samples result according to Piper and Gibbs diagram.

To accept and comprehend various hydrogeochemical variables that regulate the draining water quality in the examined area, in order to analyze hydrogeochemical data, graphical techniques like the diagrams of Piper and Gibbs were used. The geochemical water types were Ca-HCO_3_, Na- HCO_3_, Ca-Mg-Cl-SO_4_, and Na-Ca-HCO_3_ based on the chemical characteristics of the tested samples (Fig. 2). According to Piper diagram, two types of water including Ca-Mg-HCO_3_ and Ca-Mg-SO_4_ were presented based on the chemical analysis of water samples. By graphing the ratios of Na/ (Na + Ca) and Cl/ (Cl + HCO_3_) vs. TDS on Gibb’s diagram, evaporation and rock-dominated processes were the most important mechanism that effect on the chemical composition of drain water (Fig. 2a, b).

**Fig. 3:**
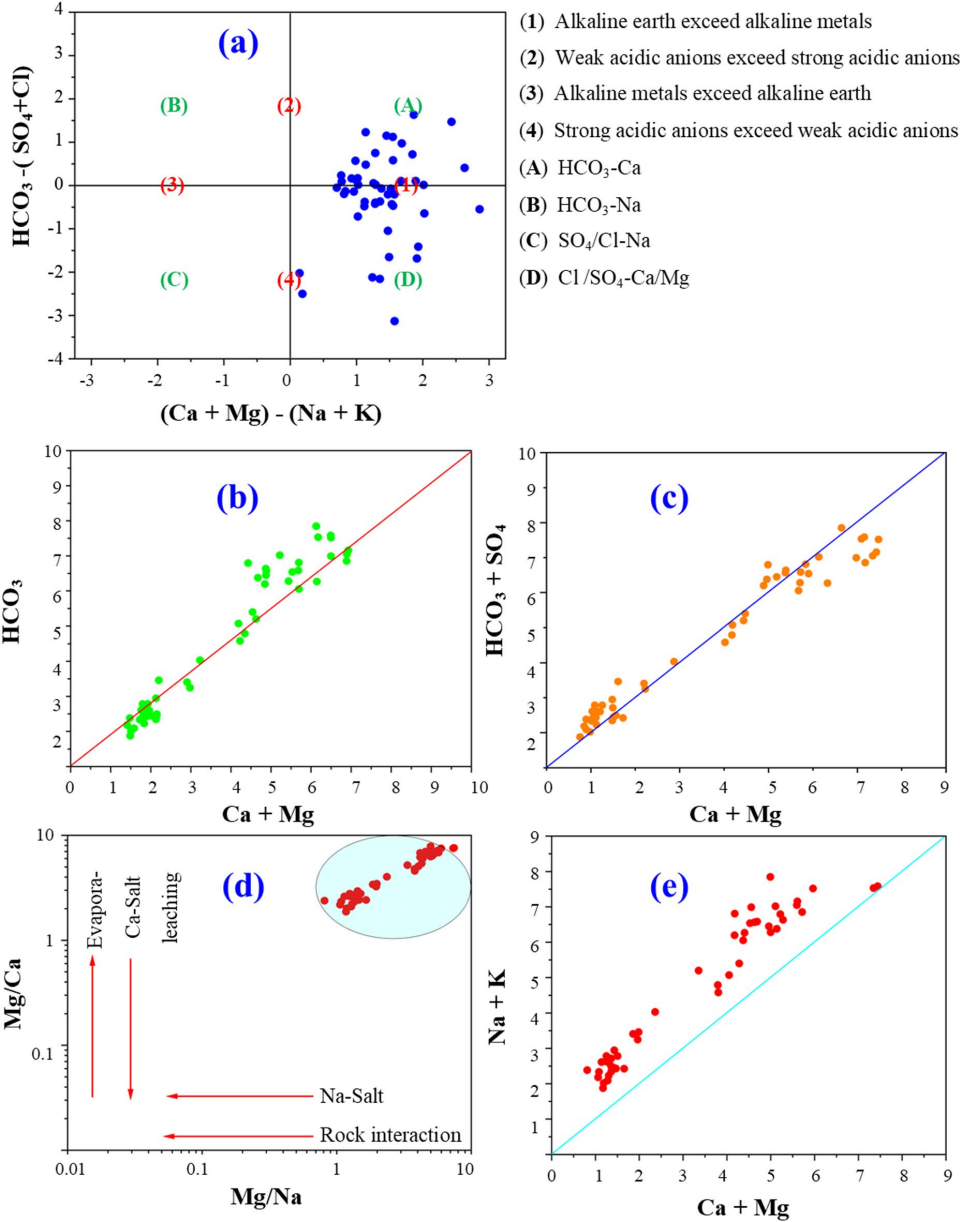
Illustration of water facies and geochemical control mechanisms (Chadha 1999).

To determine the hydrogeochemical processes and drain water facies, Chadha’s categorization was applied to the samples of collected drain water (Fig. 3). In the field (A, D), drain water samples indicated mixing with alkaline soil exceeds alkaline types (Ca- Mg- HCO_3_ and Na-HCO_3_ water facies) under the impact of rock-water interaction.

### Multiple irrigation water quality indices

The indicators, which are based on a number of water’s physiochemical characteristics, include IWQI, SAR, %Na, RSC, PI, MH, PS, KI, and PI. The irrigation water quality indices (IWQIs) were calculated according to arithmetical formulas of selected physiochemical parameters and the measurements included electrical conductivity, cations (Na^+^, K^+^, Ca^2+^, Mg^2+^), anions (HCO_3_^–^, Cl^–^, SO_4_^–2^), and other variables., which used the value of five parameters in this study. The determination of the IWQIs, including the SAR, %Na, RSC, PI, MH, PS, and KR, is dependent on a number of physiochemical water factors. The IWQIs were calculated according to arithmetical formulas of selected physiochemical parameters such as Cations (Na^+^, K^+^, Ca^2+^, Mg^2+^) and anions (HCO_3_^–^, Cl^–^, SO_4_^–2^). In addition, the statistical analysis of different IWQIs was presented in Table 8.

**Table 8:** Summary of the descriptive statistics of the determined IWQIs.

Parameter	Min.	Max.	Mean
IWQI	44.98	99.89	81.36
SAR	0.64	3.35	1.74
Na %	25.41	49.53	39.17
RSC	– 2.37	0.03	– 0.82
PI	58.16	78.46	66.90
MH	16.53	46.54	36.27
PS	1.38	7.72	3.60
KI	0.29	0.86	0.56

#### Irrigation water quality index (IWQI)

**Fig. 4:**
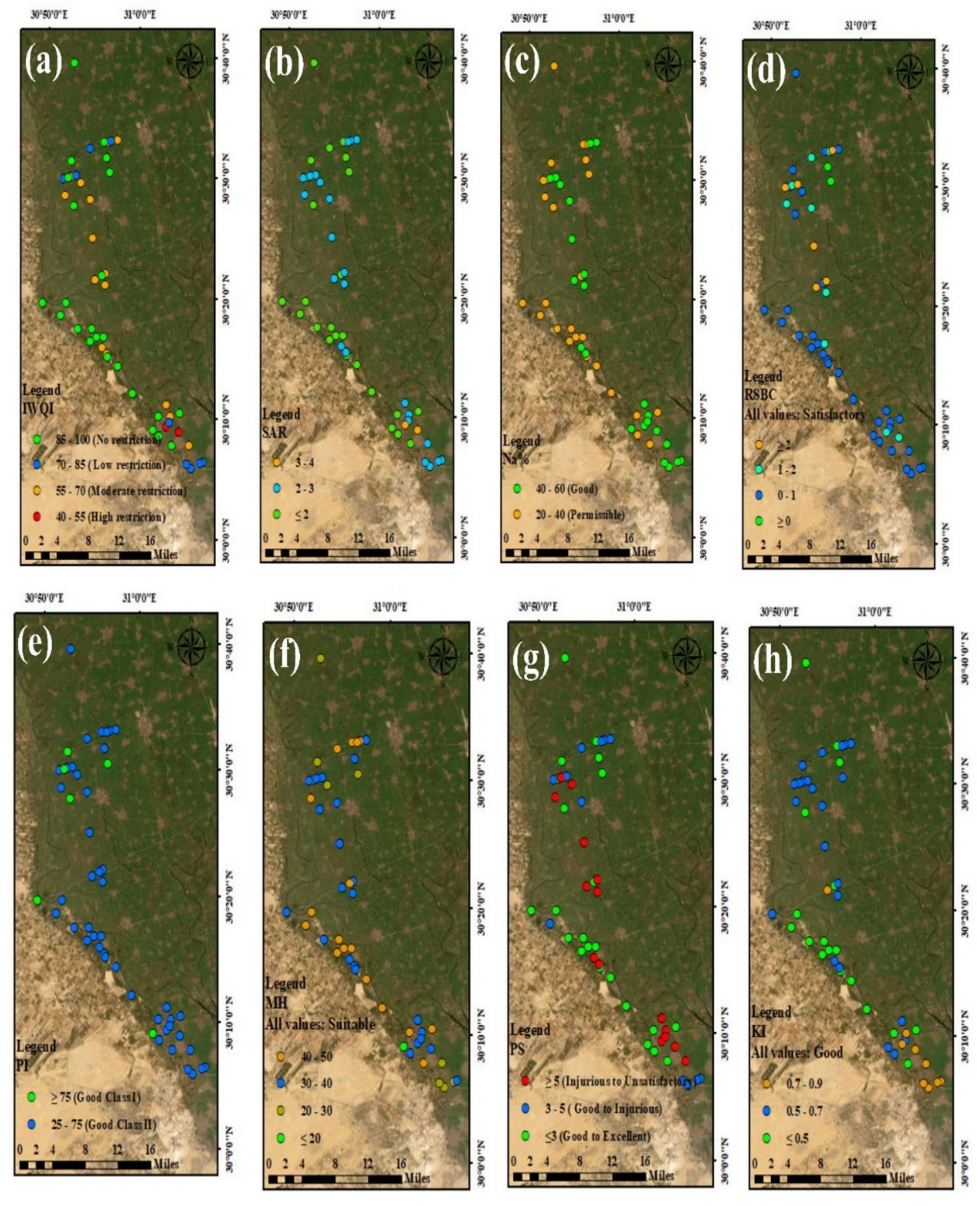
IWQIs variation maps in the research area. Map created using ArcGIS Pro 2.8.8 (Esri; https://www.esri.com/arcgis/about-arcgis).

The IWQI is a calculated index with no dimensions that reflects all the parameters affecting overall water quality. It creates a single value from several chemical parameters of water samples, offering a complete assessment of the overall water quality and suitability for agricultural irrigation^58,59^. According to the calculated IWQI values, the IWQI varied from 44.98 to 99.89, average of 81.36 as shown in (Table 8; Fig. 4a). About 4% of drain water samples have high restrictions for irrigation use 30% of samples have moderate restrictions and 18% of samples have low restrictions and 48%of sample no restriction. The maps can be used to evaluating the overall effectiveness of drainage systems and determining the suitability of water quality for irrigation.

#### Sodium adsorption ratio (SAR)

According to Suarez^60^, when evaluating whether water is suitable for irrigation, the SAR is a valuable indicator. In line with the SAR classification (Table 8), all drain samples were excellent for irrigation with a SAR less than 10, and the SAR averaged 1.74, with SAR values ranging from 0.64 to 3.35 (Table 8). Since it is a sign of an alkali or salt threat to crops, sodium concentration is a crucial factor in classifying water for irrigation. The quality of water in the research around the Rosetta Branch has declined, as seen by the SAR distribution map (Fig. 4b).

#### Sodium percentage (Na %)

Water’s sodium content reacts with the soil to decrease its permeability, which has an impact on irrigation. Na (%) is broadly used to highlight the appropriateness of water for agriculture. Generally, surface water with high Na^+^ content reacts with the soil and decreases its permeability in comparison to Ca^2+^ and Mg^2+^ concentrations. This weakens the structure of the soil and increases the number of reduced plants. According to Na% readings, most water samples (50%) was in good condition, and the remaining percentage was permissible for use in irrigation^61^. 50% of the water samples fell within an acceptable appropriateness for agricultural irrigation purposes, while the remaining 50% were in an excellent class for irrigation as shown in Table 8; Fig. 4C. The process of exchange between sodium in water and calcium and magnesium in soil significantly reduces the permeability of agricultural soil. The obtained Na% values ranged between 25.41 and 49.53, with a mean of 39.17 (Table 8).

#### Residual sodium carbonate (RSC)

The RSC has estimated the risk that carbonates and bicarbonates pose to agricultural water’s consistency. According to USEPA^62^, water is considered good for irrigation when the RSC value is less than 1.25 and unsuitable when it rises to > 2.5. RSC is produced when the alkalinity concentration is above that of the alkaline earth ions (Ca^2+^ and Mg^2+^). The RSC values revealed a mean value of -0.82, which ranged from − 2.37 to 0.03. All drain water samples were classified as excellent (100%) by the RSC results.

As shown in Table 8; Fig. 4d, the RSC values are less than 1.25. Surface water appropriateness is determined more by the effects of high alkalinity than by the total quantities of Ca^2+^ and Mg^2+^ in the water. According to Sudhakar and Narsimha^63^, The irrigation water is largely affected by two key factors: the concentration of sodium (Na⁺) and the level of alkalinity relative to alkaline earth elements. High sodium content and excessive alkalinity can significantly affect water quality, impacting its usability for agricultural purposes. Covering the soil’s pores with the salt created by continual RSC use above 2.5 meq/L can block the flow of water and air in the soil. The RSC results showed that all drain water samples were satisfactory (100%) and suitable for irrigation (Table 8; Fig. 4d). Surface water appropriateness is influenced by excess alkalinity more so than by the combined Ca^2+^ and Mg^2+^ concentrations in the water. Finally, the quantity of excess alkalinity, concentration of Na+, and alkaline earth components affect whether or not water is suitable for irrigation.

#### Permeability index (PI)

Introduced by Doneen^64^, the PI is used to empathize the appropriateness of irrigation water. Based on PI values, water is divided into three groups (Table 8). Class I (PI > 75%) is considered as suitable for irrigation and class II (PI = 25–75%) is considered as reasonably suitable, Class III (PI < 25%) is unacceptable^38,65^. Long-term use of irrigation water with high levels of Ca^2+^, Na^+^, Mg^2+^, and HCO_3_^–^ can negatively affect soil permeability^66^. However, as shown in Table 8, all the drain samples were categorized as suitable for agriculture or irrigation based on PI classification. The PI is a widely used metric to assess the suitability of irrigation water, particularly when it contains elevated concentrations of Na^+^, Ca^2+^, Mg^2+^, and alkalinity ions over extended periods^67^. In this study, the PI values for the drain water samples ranged from 58.16 to 78.46, with an average value of 66.90 (Table 8). Based on these values, the samples were categorized into two groups: 88% fell into Class II (good) and 12% into Class I (excellent) (Fig. 4e). These results confirm that the water quality of the drains is appropriate for irrigation purposes.

#### Magnesium hazard

The MH is known to be toxic and unsuitable for irrigation uses. As soils become more alkaline, this will have a negative effect on crop yields. High concentrations of magnesium (Mg^2+^) in irrigation water can lead to several agricultural challenges. These include increased soil alkalinity, reduced soil infiltration rates, decreased availability of phosphorus to plants, and ultimately, lower crop yields. Managing magnesium levels is therefore crucial for maintaining soil health and ensuring optimal agricultural productivity^68^. As a result, MH was suggested for the assessment of drain water for irrigation. The MH values of the drains water samples < 50 so all drains safe for irrigation, and 26% of sample 40–50, 34% of sample 30–40, 8% of sample 20–30 and 2% > 20 of sample respectively as shown in (Table 8; Fig. 4f).

#### Potential salinity (PS)

Only 30% of the drain water samples were classified as injurious to unsatisfactory, while 24% fell into the good to injurious category. However, the majority of the samples (46%) were classified as good to excellent, as shown in Table 8; Fig. 4g.

The Potential Salinity (PS) index is another key metric applied to evaluate the suitability of drains for irrigation. Generally, drain water with a PS value below 5 meq/L is considered safe^40^. In current investigation, the PS values for the drain water samples ranged from 1.38 to 7.72, with an average value of 3.60 (Table 8). These findings indicate that most of the samples are within acceptable limits for agricultural use.

#### Kelley ratio (KR)

Based on the amount of Na determined against Ca and Mg, an important parameter was added to determine the irrigation water quality, where all ion concentrations in meq/L are represented. A Kelley’s Ratio (KR) or Kelley’s Index (KI) > 1 specifies an excessive amount of sodium (Na). Water with low KR value (< 1) is considered acceptable, while a higher ratio suggests unsuitability due to elevated sodium levels. The KR value can detect the risk threaten the soil structure and plant production. The current results showed that the KR values for the drain samples fell within specific range (0.29 to 0.86) confirm that 100% of samples in good category (Table 8; Fig. 4h).

### Pollution indices

Pollution indices, counting the Cadmium Index (Cd), Heavy Metal Pollution Index (HPI), Metal Index (MI), and Pollution Index (PI), were calculated to assess the concentrations of various metals in the water. These metals included chromium (Cr), cobalt (Co), aluminum (Al), iron (Fe), manganese (Mn), cadmium (Cd), lithium (Li), lead (Pb), nickel (Ni), and zinc (Zn). The results of these calculations are summarized in Tables 9 and 10, providing a clear overview of the metal contamination levels and their implications for water quality.

**Table 9:** Statistical analysis of metal concentration in our samples.

Metals	Al	Cd	Co	Cr	Fe	Li	Mn	Ni	Pb	Zn
Min	0.091	0.001	0.001	0.018	0.016	0.002	0.042	0.005	0.007	0.041
Max	0.986	0.009	0.245	0.807	1.407	0.009	2.281	0.636	0.326	9.570
Mean	0.484	0.002	0.008	0.078	0.633	0.004	0.260	0.032	0.038	2.454
Stand. Dev	0.217	0.001	0.035	0.147	0.219	0.002	0.332	0.091	0.053	2.901

**Table 10:** Statistical analysis of HPI, MI and C_d_ during long term and short-term use for irrigation.

	Pollution indices (PIs)	HPI	MI	C_d_		HPI	MI	C_d_
Short-term use	Min	2.22	0.14	– 9.86	Long-term use	13.29	1.01	– 8.99
	Max	17.24	1.69	– 8.31		154.11	23.35	13.35
	Mean	4.91	0.48	– 9.52		30.71	4.10	– 5.90
	Stand. Dev	2.45	0.39	0.39		24.30	3.87	3.87

#### Heavy metal potential toxic index (HPI)

**Fig. 5:**
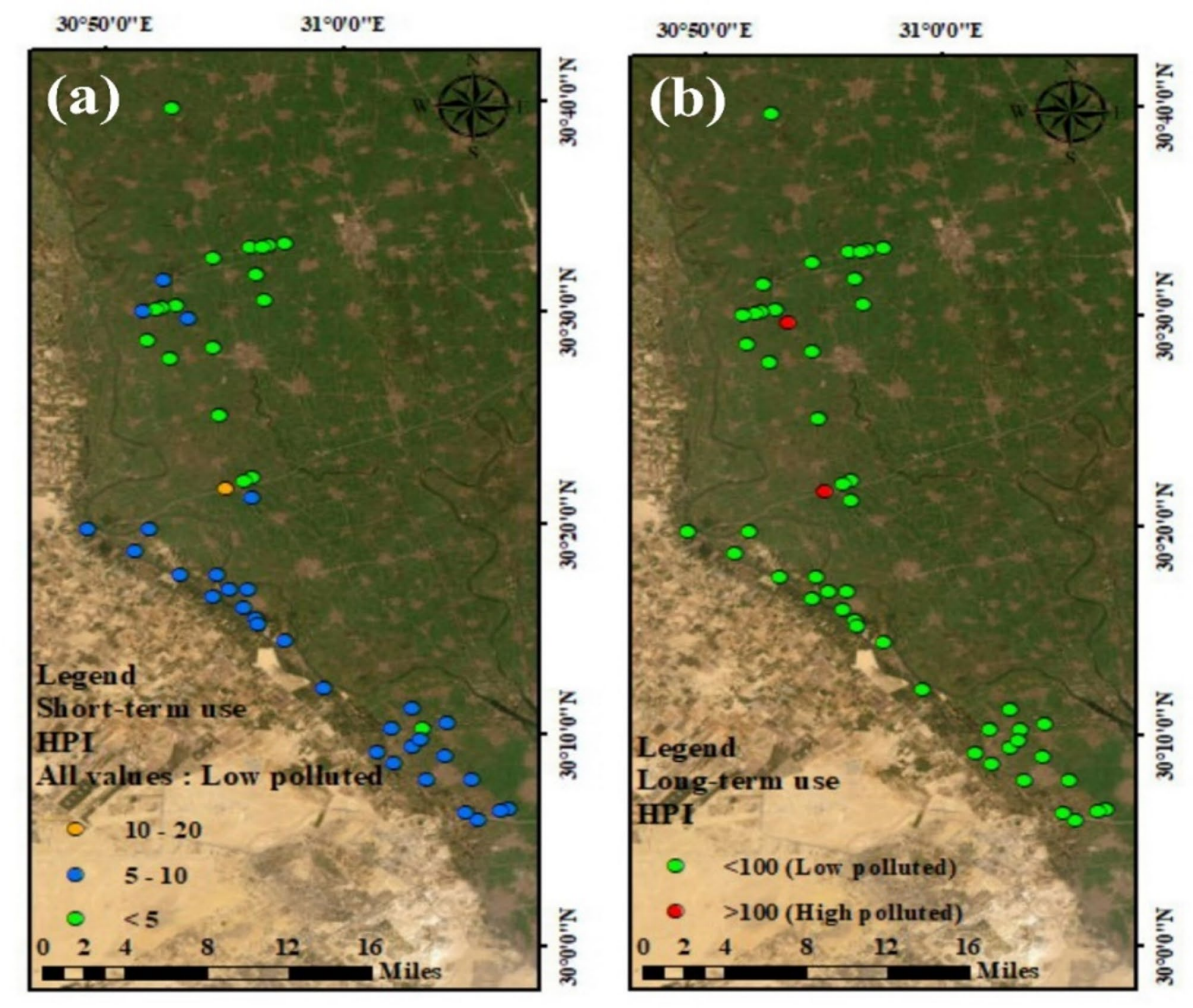
Heavy metal potential toxic index distribution maps of drains water network for short. Term (a) and long term (b) use. Map created using ArcGIS Pro 2.8.8 (Esri; https://www.esri.com/arcgis/about-arcgis).

The Heavy Metal Potential toxic index values for short-term usage varied from 2.22 to 17.24 representing that 100% of samples was below recommended value (100), indicating low pollution and suitable water for irrigation (Fig. 5a). The HPI results showed that long-term irrigation water ranged between 13.29 and 154.11, with an average of 30.71, which showing about 96% of our samples fell under the recommended value and 4% of samples above the recommended value (100) (Table 10; Fig. 5b). During long-term use, the metals had increased concentrations of Zn, Fe, Al, Mn, and Cr, which cause water contamination in two drains.

#### Metal index (MI)

**Fig. 6:**
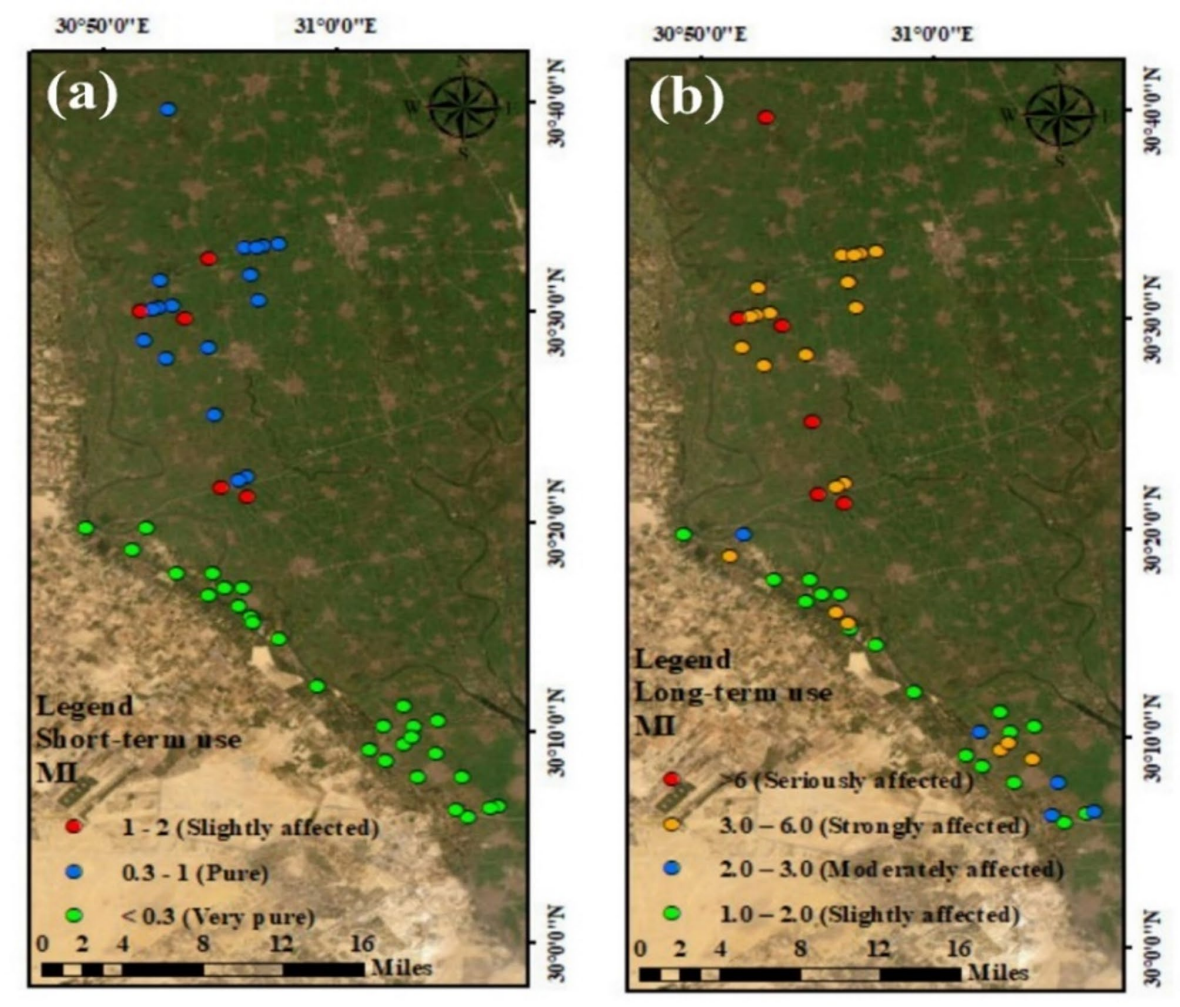
Metal index (MI) distribution maps of drains water network for short –term (a) and long – term (b) use for irrigation. Map created using ArcGIS Pro 2.8.8 (Esri; https://www.esri.com/arcgis/about-arcgis).

The MI result indicated that metals slowly impacted on the drain water samples taken. According to the MI values, 36% of samples had modest effects, 10% had moderate effects, 42% had strong effects, and 12% had significant impacts on irrigation water quality. In the long-term irrigation water use, the MI had a median value of 4.10, which ranged from 1.01 to 23.35 (Table 10). According to the statistical chemical analysis of drainage system (Fig. 6b), the top portion of Sabal drainage was revealed the activity of industry wastewater, as shown by the spatial variation map of MI results (Table 10; Fig. 6). The MI values revealed that about 58% of samples were very pure for short-term usage, while 32% of the samples were pure, and 10% of samples were slightly contaminated with metals (Fig. 6a).

#### Contamination index (C_d_)

**Fig. 7:**
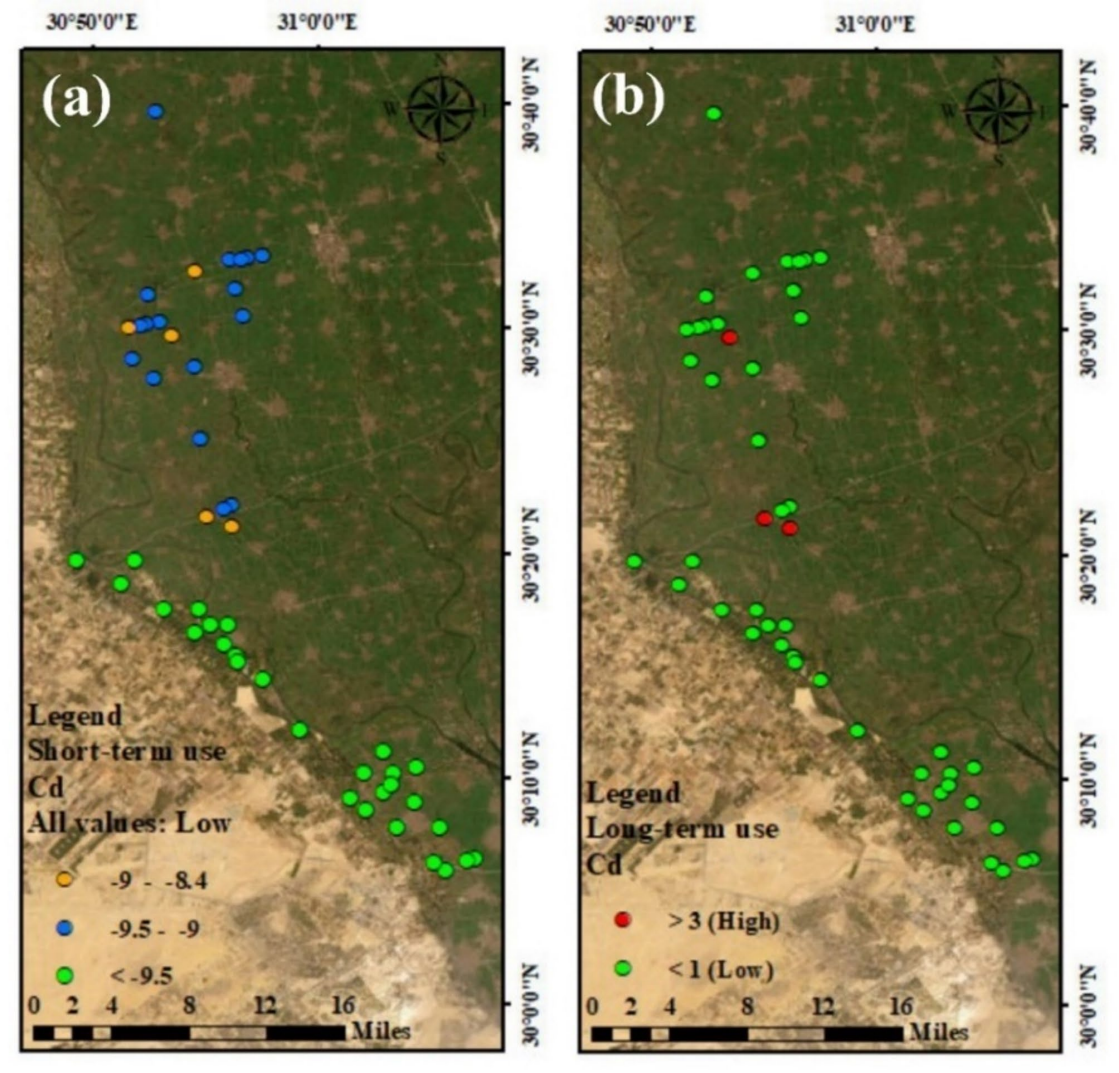
Contamination index (C_d_) distribution maps of drains water network, for short term (a) and long term (b). Map created using ArcGIS Pro 2.8.8 (Esri; https://www.esri.com/arcgis/about-arcgis).

The C_d_ levels for the water samples from the investigated drainage ranged between − 8.99 and 13.35, with an average of – 5.9 (Table 10) and (Fig. 7b) In the case of a short-used period of time. 100% of our drain water samples had C_d_ levels with negative values, which can use for irrigation (Fig. 7a). and most of the drains water samples (94%) were low contaminated with metals, while about 6% of the samples were high contaminated water over a long period of time.

#### Potential toxic index (PI)

The following metals were found to be present in the collected water samples in order of their mean concentrations in mg/L, as the following: Zn (2.454), Fe (0.633), Al (0.484), Mn (0.260), Cr (0.078), Pb (0.038), Ni (0.032), Co (0.008), Li (0.004), and Cd (0.002) (Table 11). The impact of pollution on drain water was evaluated by analyzing metal concentrations using the PI. This index provides a comprehensive measure of metal contamination, helping to assess the overall quality and suitability of the water for various uses. The five classes of data indicate the various contamination effects of each metal on the quality of the drains’ water (Table 11). All heavy metal as mean value does not affect on plant for using this water in irrigation using except Zn metal is effect on soil and plant but is moderating effect, so all drains in irrigation site can be reuse for irrigation with not effecting.

**Table 11:** The table provides an evaluation and classification of the water in the study area, focusing on the impact of toxic metals.

Metal	PI	Class	Effect
Cd	0.002	1	No effect
Li	0.004	1	No effect
Co	0.008	1	No effect
Ni	0.031	1	No effect
Pb	0.038	1	No effect
Cr	0.078	1	No effect
Mn	0.26	1	No effect
Al	0.484	1	No effect
Fe	0.633	1	No effect
Zn	2.454	3	Moderately affected

Based on the recommended maximum trace element concentrations for long-term usage established by Ayers and Westcot^69^, the PI was used to assess the quality of water (of the drains) for long-term irrigation and to estimate the toxicity and pollution resulting from trace elements (Al, Cd, Cr, Co, Li, Fe, Mn, Ni, Pb, and Zn) on plants. According to El-Amier et al.^48^, anthropogenic activities increase pollution, increase metal levels in ecosystems, and put human health in danger. According to (Table 12) most of trace element concentrations were high level of contamination and should be removed before being used for irrigation. The long-term (maximum concentration) is determined by applying water at a rate of 10,000 m^3^ per acre per year, which is consistent with good irrigation methods. In case the rate of water application is significantly higher than this, the maximum concentrations ought to be reduced correspondingly. For application rates less than 10,000 m^3^ per acre annually, no changes should be made. The values provided are for continuous water use at a single location.

**Table 12:** Recommended upper limits for trace element concentrations in irrigation water, established by the National academies of science and engineering^70^.

Trace element	Recommended maximum concentration (mg/L)		Maximum concentrations of trace elements in drains water (mg/L) in study area	
	Standard for short-term use	Standard for long-term use	PI for short term use	PI for long term use
Al	20.0	5.0	0.02	0.10
Cd	0.05	0.01	0.09	0.45
Cr	1.0	0.10	0.40	4.04
Co	5.0	0.05	0.02	2.45
Fe	20.0	5.0	0.04	0.14
Pb	10.0	5.0	0.02	0.03
Li	2.5	2.5	0.00	0.00
Mn	10.0	0.2	0.11	5.70
Ni	2.0	0.2	0.16	1.59
Zn	10.0	2.0	0.48	2.39

### The performance of spectral indices to estimate water quality indices

**Fig. 8:**
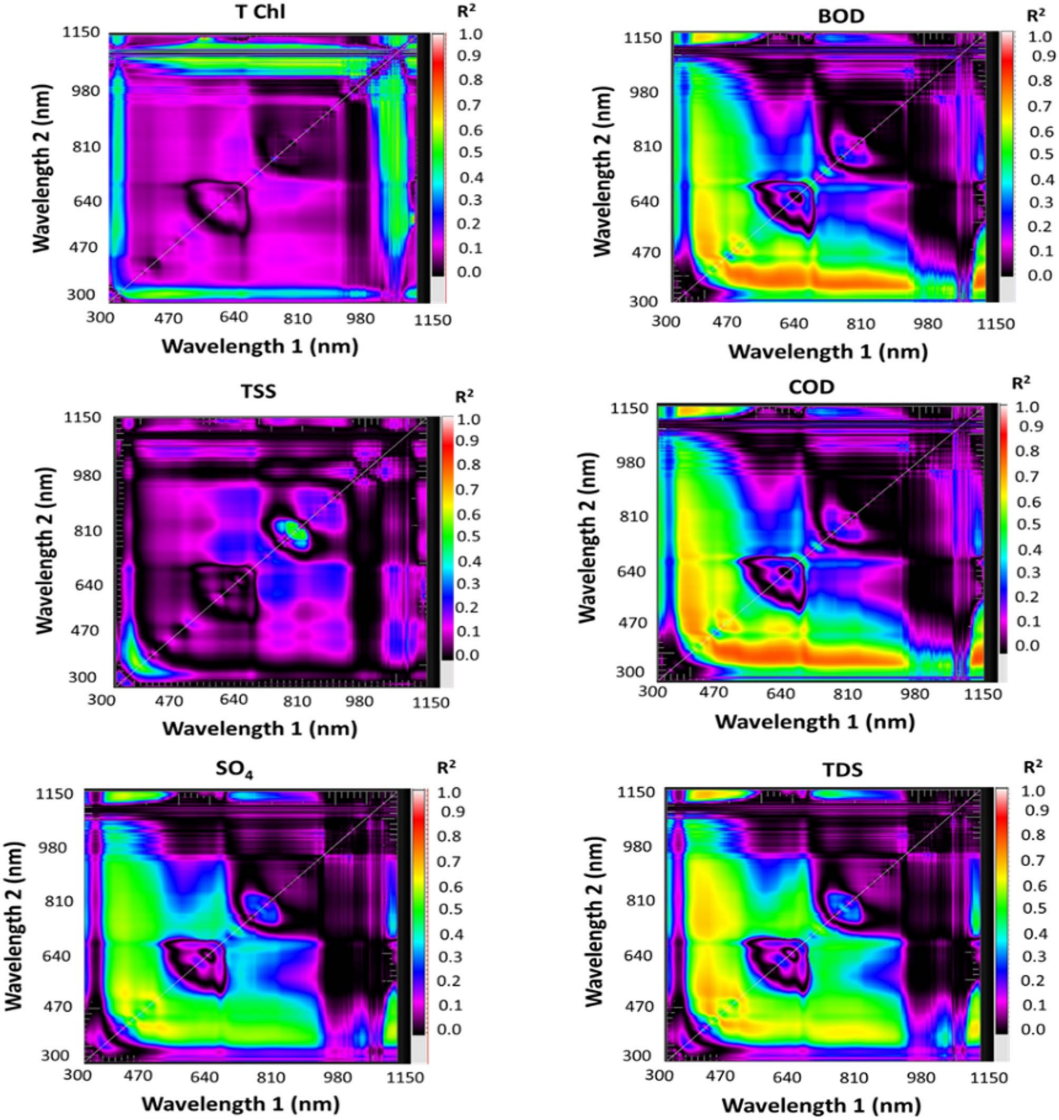
Correlation matrices displaying estimated R^2^ values for all potential two-band spectral combinations with T Chl., TSS, SO_4_, BOD, COD, and TDS of water samples.

**Fig. 9:**
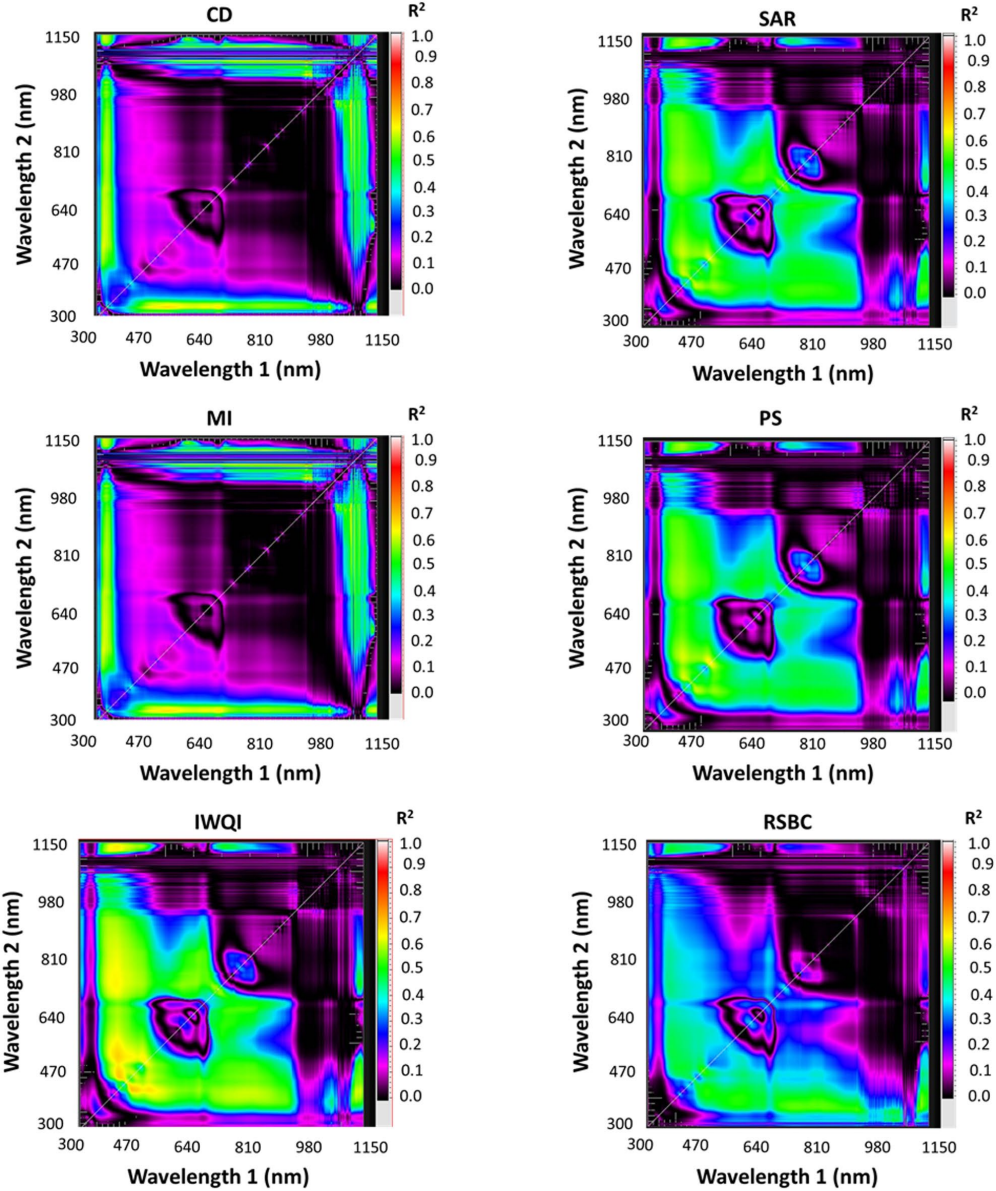
Correlation matrices displaying estimated (R^2^ values for all potential two-band spectral combinations with two pollution indices such as C_d_ and MI in long term as well as with four irrigation water quality indices IWQI, SAR, PS, and RSC of water samples.

Using the spectral reflectance from 50 drain samples, contour maps were developed to visualize the relationships between two-band spectral combinations and key water quality parameters. These parameters included Total T Chl., TSS, SO4, BOD, COD, TDS, two pollution indices such as C_d_ and MI in long term as well as with four IWQI_S_ such as IWQI, SAR, PS, and RSC. The maps illustrate the R^2^ values for these relationships, as well as the Spectral Reflectance Indices (SRIs) resulting after combinations of wavelengths with 302–1148 nm spectral range (Figs. 8 and 9). The newly developed SRIs were generated using 2-D correlograms or contour maps based on spectral reflectance data. Multiple contour maps were created using linear equations to display the coefficients of determination (R^2^) between all dual-wavelength combinations and each WQI (Figs. 8 and 9). These maps provide a comprehensive visual representation of the spectral relationships and their predictive potential for water quality assessment. The highest R^2^ hotspot area indicated the strongest correlation between SRIs and quality indices.

The specified SRIs (Table 13) were determined utilizing spectral data of water quality indices in the VIS at multiple bands and NIR bands. For example, RSI_566, 1140_ and RSI_564, 1140_ were strongly related to T Chl with R^2^ = 0.73, and RSI_456,422_ was strongly related to IWQI with R^2^ = 0.67. As well as RSI_500, 400_ had good relationship with BOD and COD with R^2^ = 0.75 and 0.78, respectively. The T Chl was detected at peak wavelengths of TSS and 1140 nm and 564–566 nm. The water samples spectrum curves showed several significant absorbance regions at 788 nm, 780 nm, 950 nm, and 980 nm beside low absorbance zone (850 nm, 452 nm, and 703 nm).

On the other hand, Gad^55^ found that SRIs were ineffective in assessing both the Cadmium Index (Cd) and the Heavy Metal Pollution Index (HPI). Their results indicated minimal pollution levels and high metal purity, making these indices less relevant for such evaluations. However, SRIs proved useful for assessing the Metal Index (MI) across varying pollution levels.

Field-measured spectroscopy has emerged as a practical and cost-effective method for large-scale, integrated monitoring of water quality. It addresses the limitations of traditional Irrigation Water Quality Indices (IWQIs) by capturing temporal and spatial changes in water quality parameters. This approach leverages a wide range of spectral reflectance bands, which are highly sensitive to variations in key physiochemical properties such as salinity, total dissolved solids (TDS), total alkalinity (TA), total hardness (TH), total suspended solids (TSS), total chlorophyll, nitrogen and phosphorus levels, dissolved organic carbon, biological oxygen demand (BOD), and chemical oxygen demand (COD).

For instance, strong correlations have been observed between SR wavelengths ranges from 450 nm to 510 nm and from 500 nm to 600 nm for the blue and green respectively and the total phosphorus content in water^71^. Similarly, Gitelson^72^ demonstrated that SR from 700 nm to 900 nm is more sensitive to variations in TSS levels. Liu^73^ further highlighted SR from 560 to 780 nm is effective for detection of suspended particles. Xing^23^ identified specific spectral response peaks for various water quality parameters. From 990 nm to 999 nm specified for ammonia-nitrogen, from 760 nm to 770 nm specified for BOD, and from 460 nm to 475 nm for TA, TH, COD, and TDS. Despite these advancements, the large volume of spectral reflectance data generated requires careful analysis using appropriate statistical methods. This step is crucial for establishing robust relationships between IWQIs and spectral data, ensuring accurate and reliable water quality assessments.

There were moderate and lower relationships between SRIs and TSS and RSS respectively. This is due to the concept underlying these techniques is that the numerous sensors in these instruments can determine changes in the water surface’s optical propertiesat various bands. The changes in the physiochemical, biological, and hydrological aspects of the water are intricately related to the water surface’s optical properties. As a result, the spectra emitted by the water’s surface could be used directly or indirectly to estimate WQIs and the relationships could be ranged from low to stong level.

**Table 13:** Relationships of linear regression of twelve indicators such as T Chl., TSS, SO_4_, BOD, COD, TDS, C_d_, MI, IWQI, SAR, PS, and RSC of water samples with several SRIs of squash expressed as determination coefficients.

SRIs	T Chl.	TSS	SO_4_	BOD	COD	TDS	C_d_	MI	IWQI	SAR	PS	RSC
RSI_566, 1140_	0.73***											
RSI_564, 1140_	0.73***											
RSI_788, 780_		0.56***										
RSI_786, 780_		0.58***										
RSI_508, 410_			0.65***									
RSI_504, 492_			0.63***									
RSI_486, 400_				0.76***	0.78***							
RSI_500, 400_				0.75***	0.78***							
RSI_462, 508_						0.70***						
RSI_522,424_						0.70***						
RSI_874,1072_							0.78***	0.78***				
RSI_1072,920_							0.71***	0.71***				
RSI_456,422_									0.67***	0.60***	0.61***	0.48***
RSI_420,458_									0.67***	0.60***	0.61***	0.48***

*** Significant at *p* ≤ 0.001 probability levels.

#### Prediction of different WQIs using PLSR

**Fig. 10:**
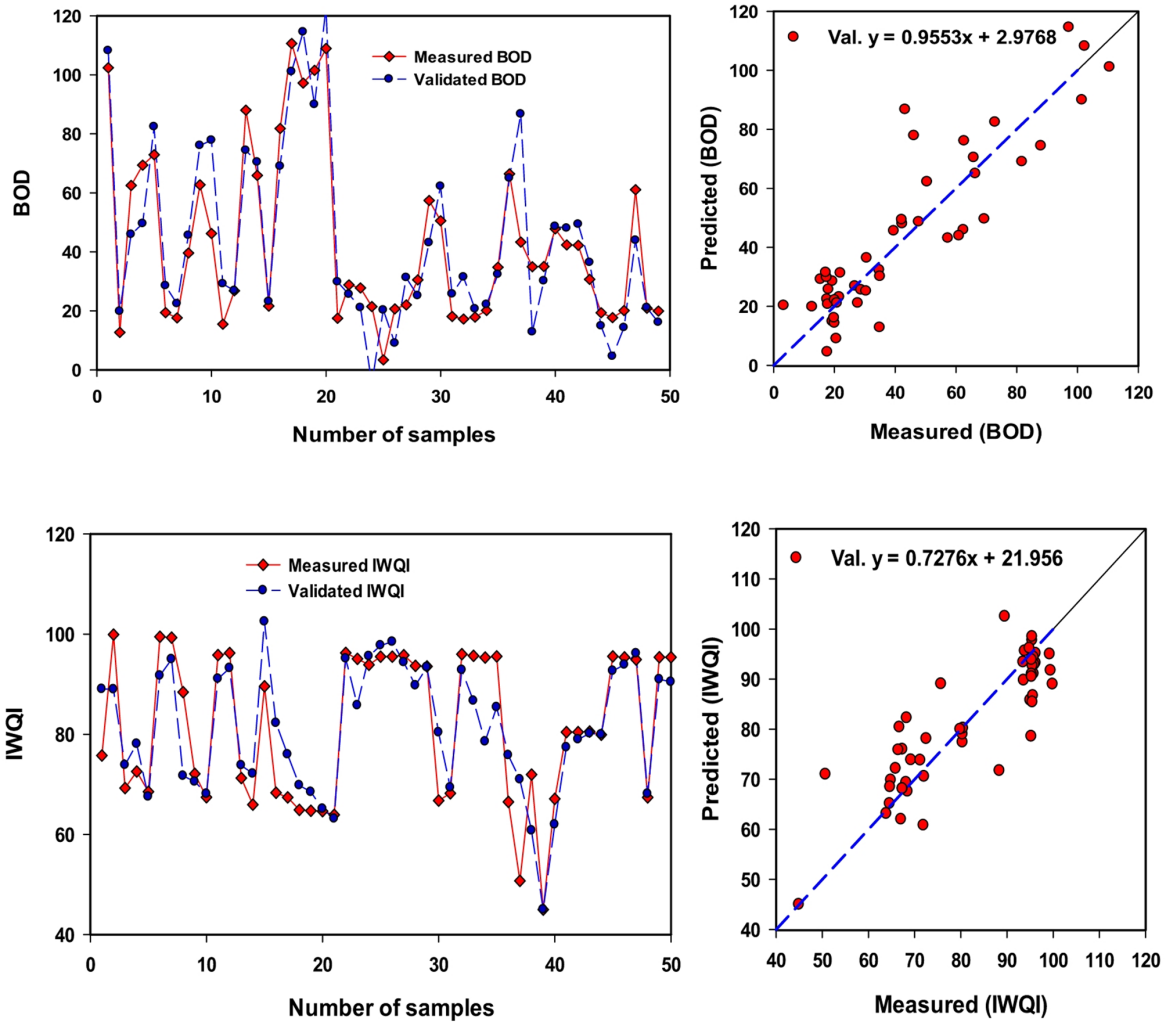
Comparison between measuring datasets, and validating datasets for BOD, and IWQI of water samples.

SRIs offer a straightforward and efficient method for assessing water quality, making them ideal for developing compact, lightweight spectral sensors. These sensors can be used for rapid, cost-effective, and large-scale water quality monitoring. However, each SR indicator typically concentrations on 2 or 3 sensitive wavebands, which limits their ability to handle complex scenarios. For example, significant variations in levels of water components, and saturation levels measurements, can complicate the development of effective SRIs. To address these challenges, this study employed Partial Least Squares Regression (PLSR) models, using SRIs as input variables to predict Irrigation IWQIs (Fig. 10). Overall, the PLSR models demonstrated higher accuracy in estimating IWQIs. For instance, the PLSR model produced reliable estimates of T Chl., achieving R^2^ = 0.87 and 0.77 for the calibration and validation dataset. Similarly, the model provided accurate predictions for BOD, with R^2^ = 0.96 and 0.81 for calibration and validation (Table 14; Fig. 10).

Gad et al.^55^ found that in Val. datasets, the PLSR model generated strong estimates for the DWQI and MI with R^2^ of 0.82 and 0.79 and RMSE of 20.82 and 2.77, respectively, for the Rosetta branch and with R^2^ of 0.93 and 0.78 and RMSE of 11.67 and 2.22, respectively, for the Damietta branch. However, the PLSR model provided moderate estimation performance for Turb. and TSS in the Val. datasets, with R2 of 0.55 and 0.62 and RMSE of 1.18 and 6.60, respectively. Wang et al.^74^ discovered that detecting TSS in moderately clear water using a single band or two waveband combinations is difficult. However, in turbid water bodies, a combination of three wavebands proved successful in estimating Chl-a levels^50^.Wang^75^ also highlighted that the models incorporating a significant wavebands number outperformed those relying on only one or two wavebands when predicting inland water quality indicators. This underscores the potential of PLSR models, combined with multiple SRIs, as a unified approach for remote water quality assessment. By leveraging these models, researchers and practitioners can achieve more robust and reliable evaluations of water quality across diverse conditions.

**Table 14:** Results of PLSR calibration and validation models for relationship between SRIs and T Chl., TSS, SO_4_, BOD, COD, TDS, C_d_, MI, IWQI, SAR, PS, and RSC of water samples.

Parameters	Calibration		Validation	
	*R* ^2^	RMSE	*R* ^2^	RMSE
T Chl.	0.87***	2.95	0.77***	3.12
TSS	0.94***	9.65	0.80***	15.71
SO_4_	0.90***	9.39	0.81***	12.45
BOD	0.96***	5.89	0.81***	13.2
COD	0.87***	18.74	0.65***	33.33
TDS	0.99***	31.37	0.75***	149.89
C_d_	0.83***	0.16	0.76***	0.185
MI	0.88***	0.13	0.74***	0.19
IWQI	0.90***	5.44	0.74***	7.56
SAR	0.89***	0.27	0.74***	0.38
PS	0.88***	0.73	0.74***	0.98
RSC	0.40***	0.66	0.37***	0.61

*** Statistically significant at *P* ≤ 0.001.

### Pollution mitigation strategies

**Source control:** Reducing industrial and agricultural runoff through stricter regulations (e.g., enforcing limits on heavy metal discharges, as noted in Table 12).

**Treatment technologies:** Implementing cost-effective methods (e.g., constructed wetlands for nutrient removal, adsorption filters for metals like Zn/Mn) in high-risk drains (e.g., Sabal drainage, where MI indicated industrial impact; Fig. 6b).

**Farmer engagement:** Promoting blended irrigation (mixing drain water with freshwater) for samples with high IWQI restrictions (4%) or elevated Zn (PI = 2.45).

**Monitoring:** Adopting spectral tools (e.g., RSI456,422 for IWQI) for real-time quality checks, as demonstrated by our PLSR models (R^2^ = 0.74 for validation; Table 14).

## Conclusion

This study comprehensively evaluated the suitability of wastewater for irrigation in Egypt’s Nile Delta region through an integrated approach combining conventional water quality indices, hyperspectral reflectance measurements (302–1148 nm), and multivariate statistical modeling. Analysis of 50 drain water samples revealed Ca-Mg-HCO_3_/SO_4_ as dominant hydrochemical facies, with rock-water interactions and evaporation identified as key governing processes. The IWQI classification indicated that 66% of samples were suitable for irrigation, while 4% posed high-risk due to elevated salinity and localized metal contamination (e.g., Zn with PI = 2.45). Crucially, the study developed novel spectral reflectance indices (RSI566,1140 for chlorophyll prediction, R^2^ = 0.73; RSI456,422 for IWQI estimation, R^2^ = 0.67) and demonstrated the robust predictive capability of PLSR models, particularly for BOD (validation R^2^ = 0.81) and total chlorophyll (R^2^ = 0.77), highlighting their potential for rapid water quality monitoring. However, limitations include the moderate sample size (*n* = 50), which may affect model generalizability, and inherent challenges in spectral measurements under variable atmospheric conditions. Future work should focus on upscaling these techniques through satellite-based hyperspectral systems, testing mitigation strategies (e.g., adsorption filters for Zn-rich drains), and developing cost-effective spectral sensors for farmer-led water quality assessment. By bridging traditional water quality analysis with advanced remote sensing tools, this research provides an actionable framework for sustainable wastewater reuse in water-scarce agricultural regions, supporting both environmental conservation and food security objectives.

## Data Availability

All data are provided as tables and figures.
